# Extracellular Vesicles and PlantCrystals for Improved Bioavailability of Curcumin as a BCS Class IV Drug

**DOI:** 10.3390/molecules29245926

**Published:** 2024-12-16

**Authors:** Muzn Alkhaldi, Tehseen Sehra, Soma Sengupta, Cornelia M. Keck

**Affiliations:** 1 Department of Pharmaceutics and Biopharmaceutics, Philipps-Universität Marburg, Robert-Koch-Str. 4, 35037 Marburg, Germany; muzn.alkhaldi@pharmazie.uni-marburg.de (M.A.); tehseen.sehra@pharmazie.uni-marburg.de (T.S.); masogh@gmail.com (S.S.); 2 Institute of Pharmacy, Faculty of Pharmaceutical and Allied Health Sciences, Lahore College for Women University, Lahore 54000, Pakistan

**Keywords:** soybeans, extracellular vesicles, exosomes, differential ultracentrifugation, plantCrystals, bead milling, dermal drug delivery

## Abstract

The limited water solubility of active compounds remains a significant challenge for efficient dermal drug delivery, particularly for BCS class IV drugs such as curcumin. This study aimed to enhance curcumin’s dermal penetration using two strategies: extracellular vesicles (EVs) and plantCrystals derived from soybeans. EVs were isolated using classical methods. However, plantCrystals containing extracellular vesicles (PCEVs) were formed during the preparation of plantCrystals through bead milling. Curcumin was either added after PCEVs were formed, resulting in curcumin-added PCEVs, or added to the soybean dispersion before bead milling, forming curcumin-loaded PCEVs. The formulations were characterized for their physicochemical properties and assessed for dermal penetration efficacy using quantitative dermatokinetic and semi-quantitative ex vivo porcine ear models. The results indicated that curcumin-loaded PCEVs achieved higher penetration efficacy compared to curcumin-added PCEVs and curcumin-loaded EVs, with approximately 1.5-fold and 2.7-fold increases in penetration efficacy, respectively. Additionally, curcumin-loaded PCEVs showed superior penetration depth, while curcumin from the curcumin-loaded EVs remained in the stratum corneum. These findings suggest that the plantCrystals strategy via bead milling offers a more effective approach than the classical EVs strategy for improving the topical delivery of class IV drugs like curcumin.

## 1. Introduction

Extracellular vesicles (EVs) are heterogeneous cell-derived vesicles that can be classified into different subtypes based on their size, biogenesis, or molecular composition. Among these, small EVs represent one of the subtypes of EVs and are defined by a size of less than 200 nm [1,2]. Exosomes, a well-known example of small EVs, are lipid bilayer vesicles secreted by various cell types through inward budding of the endosomal membrane and released upon fusion of multivesicular bodies with the plasma membrane [3,4]. EVs, particularly exosomes, are able to meditate the cell-to-cell communication and serve as delivery vehicles for water-soluble and non-water-soluble therapeutic agents [2,3,5,6], which enables them to be used as drug delivery nanocarriers [6,7,8,9,10,11].

Plant-derived EVs have been recognized for their potential biocompatibility with mammalian-derived exosomes. Additionally, their characteristics have been proven to provide prolonged bioavailability compared to artificially synthesized nanoparticles because they are undetectable by the mammalian immune system [4,7,12,13,14,15]. Moreover, researchers have investigated different isolation approaches for edible plant-derived EVs from various plant sources, such as grape, blueberry, ginger, grapefruit, soybean, black kidney, and cucumber [2,11,14,15,16,17]. Previous studies have already demonstrated that plant-derived EVs offer promising potential for efficient drug delivery through many routes of administration, including dermal routes. *Aloe saponaria* has demonstrated potential as a natural treatment for chronic wound healing, and *Cucumis sativus* L. has shown enhanced dermal drug delivery [11,12,18,19].

In addition to EVs, plantCrystals offer an innovative formulation strategy to enhance the solubility and effectiveness of plant-derived compounds. They are derived from whole plants, plant parts, or byproducts and are produced by an eco-friendly extraction process that bypasses the need for organic solvents. Wet milling techniques, such as high-pressure homogenization and bead milling, are particularly effective for extracting lipophilic and poorly water-soluble compounds and achieving particle sizes below 10 µm [20,21,22]. Previous research has reported that the use of either high-pressure homogenization, bead milling, or a combination of both, allows for the breakdown of plant cells and accelerates the plant constituent’s extraction [22]. Recent evidence also suggests that the nano-milling process not only produces plantCrystals, i.e., parts of plants of submicron size, but also produces plantCrystals extracellular vesicles (PCEVs), in which drugs and/or other plant constituents can be incorporated [18,23].

*Glycine max*, commonly known as soybean and part of the Fabaceae family, is widely recognized for nutritional and therapeutic purposes due to its rich composition of protein, fiber, fats, and beneficial phytochemicals [24,25,26]. Numerous studies have highlighted the dermatological beneficial effects of soybeans, as soybean-based treatments have been found to enhance skin hydration and elasticity [25,27,28], as well as provide a photo-protective effect [29,30]. However, no studies have investigated soybean-derived EVs and PCEVs, which have neither been produced nor explored for dermal drug delivery before. This gap has prompted our interest in evaluating their dermal efficacy as delivery systems for poorly water-soluble drugs.

Several BCS class IV drugs are suitable for assessing the effects of EVs and PCEVs on dermal delivery. Curcumin possesses a broad range of pharmacological effects, including antioxidant, anti-inflammatory, antimicrobial, and wound-healing properties. In addition, the literature suggests that curcumin has the potential to treat skin diseases like atopic dermatitis and psoriasis [31,32,33,34]. Alongside its therapeutic properties, curcumin was selected as a fitting candidate due to its intrinsic autofluorescence [31,35], which enables tracking of dermal bioavailability [36]. In this context, curcumin-loaded EVs and PCEVs appear to be considerable potential formulation strategies for enhancing curcumin’s dermal penetration efficacy.

In this study, EVs were produced using the classical isolation method, yielding classical EVs and small-scale bead milling, resulting in PCEVs. Two types of PCEVs were produced. Type I PCEVs were produced by adding curcumin to the PCEVs after their production (curcumin-added PCEVs), and type II PCEVs were produced by adding curcumin prior to the production of the PCEVs to the mixture (curcumin-loaded PCEVs). Based on a previous study, type I PCEVs were considered to contain curcumin not encapsulated in the PCEVs but outside the PCEVS, whereas type II PCEVs were considered to contain curcumin mainly localized inside the PCEVs. Based on the concept of EVs and PCEVs, it was hypothesized that the encapsulation of curcumin in EVs and PCEVs would lead to better physical stability and increased penetration efficacy of curcumin. To test this hypothesis, three different formulations were characterized based on their physicochemical properties, i.e., size, size distribution, morphology, and zeta potential. Their dermal penetration efficacy was evaluated through quantitative dermatokinetics over 24 h and semi-quantitatively by using an ex vivo porcine ear model that allowed the assessment of the total amount of curcumin that penetrated into the skin as well as the penetration depth into the skin from the different formulations.

## 2. Results and Discussion

### 2.1. Production and Characterization of Soybean-Derived EVs and PCEVs Formulations

**Figure 1:**
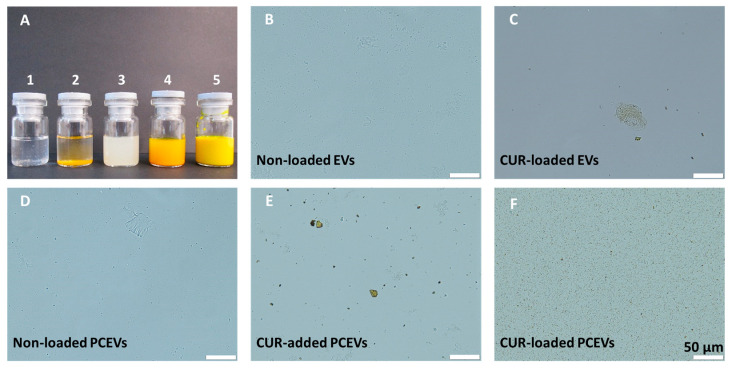
Macroscopic images of the produced formulations ((**A**); 1–5); A1: non-loaded EVs, A2: curcumin-loaded EVs, A3: non-loaded PCEVs, A4: curcumin-added PCEVs, and A5: curcumin-loaded PCEVs. Light microscopic images of the formulations (**B**–**F**) at 400-fold magnification. Curcumin (CUR), extracellular vesicles (EVs), plantCrystals extracellular vesicles (PCEVs).

Soybean-derived EVs, both non-loaded and curcumin-loaded (Figure 1; A1, A2), were successfully produced using the classical isolation method [4,15,18,37]. Additionally, non-loaded PCEVs, curcumin-added PCEVs, and curcumin-loaded PCEVs were produced (Figure 1; A3–A5). These formulations were further characterized in terms of their physicochemical properties.

Examination of particle distribution and particle size homogeneity within the formulations was conducted by light microscopy (LM) (Figure 1). LM images of curcumin-loaded EVs showed the presence of larger particle sizes after curcumin incorporation, while LM images of curcumin-added PCEVs showed a non-homogenous distribution of different particle sizes without any notable particle agglomeration. In contrast, curcumin-loaded PCEVs demonstrated homogeneity in particle distribution and relatively smaller particle sizes compared to the other formulations.

**Figure 2:**
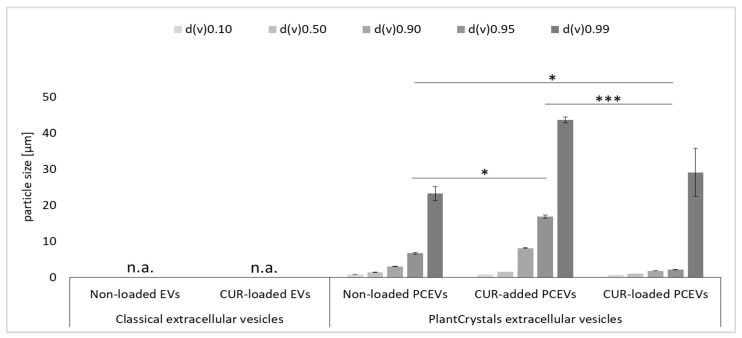
Physicochemical characterization of the produced non-loaded and curcumin-loaded EVs and non-loaded and curcumin-based PCEVs by Laser diffraction (LD). Statistical comparisons were performed on the (LD data d(v)0.95) across the formulations (curcumin-loaded EVs, curcumin-added PCEVs, and curcumin-loaded PCEVs). Significant differences between the formulations are indicated by asterisks (*: *p* < 0.05, ***: *p* < 0.001). n.a.: not applicable.

Laser diffraction (LD) was also applied to confirm the results obtained by LM, i.e., to detect any possible larger particles in the PCEVs formulations that cannot be detected by dynamic light scattering (DLS), which can only measure particles up to about 6 µm [38,39]. LD measurements provided volume-based size distribution measurements (Figure 2), where the median volumetric diameters, d(v)0.10–d(v)0.99 [µm], represent the percentages of particle volume within the dispersions that are equal to or below each given size [40]. The d(v)0.95 measurements were approximately 7 µm for the non-loaded PCEVs, 17 µm for the curcumin-added PCEVs, and 2 µm for curcumin-loaded PCEVs, showing minimal standard deviations. Moreover, a highly significant difference was observed between curcumin-added PCEVs and non-loaded PCEVs (*p* < 0.001). The detection of larger particles, especially curcumin-added PCEVs, confirmed the presence of a micro-sized range of particles. This aligns with LM images, suggesting that the addition of curcumin bulk powder to the soybean dispersion after bead milling cannot incorporate curcumin into the PCEVs. However, adding curcumin powder before bead milling enables the loading of curcumin into the PCEVs, resulting in significantly reduced particle sizes (*p* < 0.05), as seen for curcumin-loaded PCEVs.

**Figure 3:**
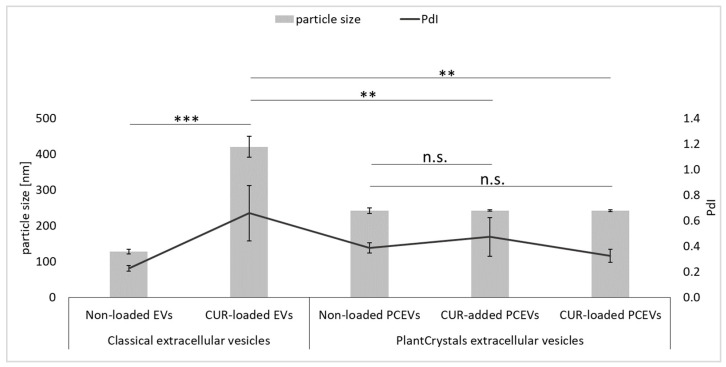
Physicochemical characterization of the produced non-loaded EVs, curcumin-loaded EVs, non-loaded PCEVs, curcumin-added PCEVs, and curcumin-loaded PCEVs by dynamic light scattering (DLS). Polydispersity index (PdI). Statistical comparisons were performed on the particle size data of the formulations (curcumin-loaded EVs, curcumin-added PCEVs, and curcumin-loaded PCEVs). Significant differences between formulations are indicated by asterisks (**: *p* < 0.01, ***: *p* < 0.001), while n.s. indicates no significant differences.

The hydrodynamic particle size of non-loaded EVs was 128 ± 7 nm with a polydispersity index (PdI) of 0.23 ± 0.02. After curcumin was loaded into EVs, the particle size increased significantly (*p* < 0.001) to approximately 421 ± 29 nm with a PdI of 0.66 ± 0.22. For PCEVs, the hydrodynamic particle size resulted in relatively similar values, with no statistical differences observed after addition of loading curcumin, indicating around 242 ± 7 nm, 243 ± 2 nm, and 242 ± 3 nm, with PdI values of approximately 0.39 ± 0.04, 0.47 ± 0.15, and 0.32 ± 0.05 for non-loaded PCEVs, curcumin-added PCEVs, and curcumin-loaded PCEVs, respectively (Figure 3).

The addition or loading of curcumin into the non-loaded PCEVs maintained the existence of nano-sized particles within both formulations and resulted in significantly lower particle sizes in comparison with curcumin-loaded EVs (*p* < 0.01). The PdI values of the formulations were higher than 0.2, indicating a broad particle size distribution and potential physical instability. To minimize any possible changes in particle size, the formulations were prepared prior to dermal penetration efficacy assessment. To enhance stability, increasing the concentration of the surfactant used is recommended. The DLS data, therefore, confirm the results from LM and LD measurements, which also showed larger sizes and broader size distributions for the curcumin-added PCEVs due to the poor incorporation of the curcumin powder into PCEVs formulations.

In the next step, the zeta potentials of the formulations were determined (Table 1). The non-loaded EVs showed a zeta potential of −21 mV, and the non-loaded PCEVs showed a zeta potential of −35 mV. However, for both types of vesicles, the zeta potential decreased upon the incorporation of curcumin by 3–5 mV, and the most pronounced decrease in zeta potential (−10 mV) was seen for the curcumin-added PCEVs (Table 1). A decrease in zeta potential in the presence of curcumin is rational, given that curcumin has a neutral charge to a slightly positive charge when ionized due to the presence of hydrogen ions [41,42]. The differences in zeta potential changes between the curcumin-loaded and curcumin-added formulations prove that curcumin is incorporated into the PCEVs, whereas curcumin seems to be more freely available outside the vesicles in the dispersion where curcumin was added to the PCEVs after their production. All formulations showed a negative zeta potential, suggesting electrostatic stabilization for all formulations produced. The highest zeta potential was found for PCEVs, which suggests that these particles can be considered to show an improved physical stability when compared to the EVs [18].

The physicochemical characterization data are in line with previous studies indicating the production of EVs using the classical isolation method and plantCrystals strategy and incorporation of the drug in the produced vesicles [18].

### 2.2. Evaluation of Dermal Penetration Efficacy of EVs and PCEVs Formulations

#### 2.2.1. Dermatokinetics

In this part of the study, the dermatokinetics profiles of curcumin from the different formulations were evaluated using ex vivo porcine ear full-thickness skin. The formulations were applied to the skin, and a fluorescence plate reader with a bottom read was utilized to efficiently determine the amount of curcumin that was able to penetrate through the skin over time. From the data obtained, penetration profiles were generated and used to compare the dermatokinetics profiles of curcumin from the different formulations [43].

**Figure 4:**
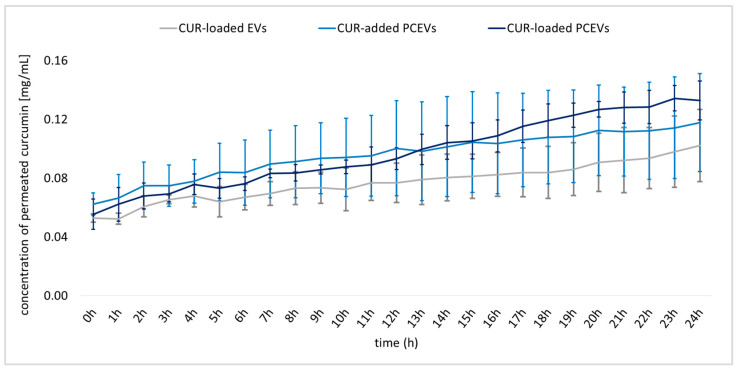
Overview of penetrated curcumin concentration [mg/mL] from the formulations using the dermatokinetic ex vivo porcine ear model.

The penetration profiles of curcumin-loaded EVs, curcumin-added PCEVs, and curcumin-loaded PCEVs exhibited a gradual increase in penetration throughout the investigated 24 h period (Figure 4). Among the formulations, curcumin-loaded EVs were found to have the lowest penetration profile. This finding suggests that EVs obtained by the classical method are less effective than PCEVs produced by small-scale bead milling. Moreover, curcumin-added PCEVs revealed slightly higher penetration than curcumin-loaded PCEVs for approximately 12 h, after which the penetration profile of curcumin-loaded PCEVs exceeded that of curcumin-added PCEVs for the rest of the investigated period.

This observation was not expected but can be explained by a mechanism that is known as particle-assisted dermal penetration [38]. Particle-assisted penetration is the increased dermal penetration of active compounds due to the presence of solid particles that can come into contact with the skin after topical application. The more particles that are in contact with the skin, and the longer the duration of the contact, the more pronounced the dermal penetration of the active ingredient.

In comparison to the curcumin-loaded EVs and curcumin-loaded PCEVs, curcumin-added PCEVs formulation is characterized by the presence of larger particle sizes besides the small-sized PCEVs. Therefore, after topical application, large curcumin particles can sediment and come into contact with the skin, thus promoting particle-assisted dermal penetration of curcumin [44]. Consequently, this formulation promotes a faster and more pronounced local concentration gradient and facilitates improved passive dermal diffusion of curcumin from curcumin-added PCEVs. However, this trend was not statistically significant (*p* > 0.5) and was overwritten by other effects, making PCEVs the most effective formulation for curcumin among all formulations tested. The results suggest that various penetration mechanisms impact the results at different time points, which, in turn, are influenced by several different parameters, such as the composition of the formulations and their physicochemical properties and the diffusion coefficient of curcumin within the formulations.

For example, EVs were the formulations with the least penetration. This formulation contained the largest particles and—due to the purification process during production, where all other materials were separated—it also contained the lowest number of particles. Curcumin can be considered to be mainly encapsulated inside the EVs. The PCEVs with added curcumin after the production, hence it was added after the PCEVs were formed, contain curcumin mainly outside the vehicle. This meanscurcumin is considered to be not encapsulated inside the PCEVs. Light microscopy and LD confirmed this and showed the presence of lager-sized curcumin microcrystals. The PCEVs with loaded curcumin can be considered to contain curcumin mainly inside the PCEVs.

As a possible interpretation of the results obtained, it can be assumed that the larger EVs with curcumin inside—due to their larger volume-to-surface ratio–release curcumin slower than smaller-sized PCEVs. This then causes a slower and less efficient penetration of curcumin when compared to PCEVs. The PCEVs with added curcumin contain curcumin microparticles. These particles are large enough to sediment onto the skin quickly after application and will thus foster particle-assisted dermal penetration (c.f. above). The particle-assisted dermal penetration effect is rapid and therefore explains the most efficient dermal penetration of this formulation at the beginning of the dermatokinetic experiment. Over time, PCEVs became the most effective formulation. This result was expected, as the formulation contains small particles with encapsulated curcumin that can release curcumin over time. In addition, these particles are considered to perform particle-assisted dermal penetration; however, due to their small size, the particles will not sediment upon application but will remain within the formulation until the liquid around the particles is evaporated. Only after this they can connect to the skin, where they will release curcumin. After they are connected to the skin, penetration is most efficient because this formulation contains the highest number of particles and the smallest particle size, thus leading to the most efficient particle-assisted dermal penetration among all three formulations. In addition to particle-assisted dermal penetration, EVs and PCEVs are also considered to foster dermal penetration via other pathways, such as passive diffusion. For this, a small particle size and efficient encapsulation of the active ingredient are needed. Therefore, if this penetration mechanism is considered, the PCEVs are also in favor, as curcumin is encapsulated, and the particles of the PCEVs are the smallest of all formulations investigated. Which penetration mechanism is most likely and dominant for the formulations investigated cannot be understood from this set of data. Therefore, more research is needed to understand this in more detail.

Previous studies have already shown the complex interplay between the formulation, active compound, and skin. This study aligns with previous studies and proves that more research is needed to understand these effects and interactions in more detail [38,45,46].

#### 2.2.2. Dermal Penetration Parameters

**Figure 5:**
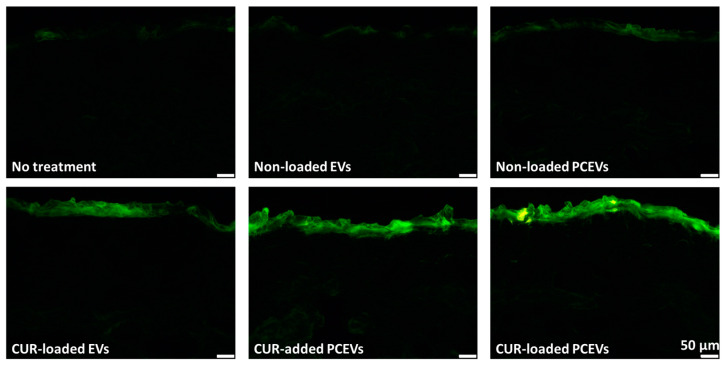
Vertical skin sections obtained by inverted epifluorescence microscopy (200-fold magnification) showing untreated skin cut, non-loaded EVs, PCEVs, and curcumin-based formulations.

Next, the dermal penetration parameters of the formulations, such as the amount of curcumin penetrated and the penetration depth into the skin, were determined. For this, vertical skin cuts from porcine skin—treated with the different formulations—were analyzed using epifluorescence images (Figure 5), along with subsequent digital image analysis [18,36].

Epifluorescence images of the skin cut upon application of the formulations showed differences in penetration efficacy for the different formulations (Figure 5). Digital image analysis of the images was then used to subtract the autofluorescence of the skin from the images with a previously established RGB threshold macro that was applied to each image [36]. The remaining autofluorescence in each image after the macro was attributed to the penetrated amount of curcumin, i.e., the penetration efficacy, that was expressed as the ART (after RGB threshold) value [MGV/px].

**Figure 6:**
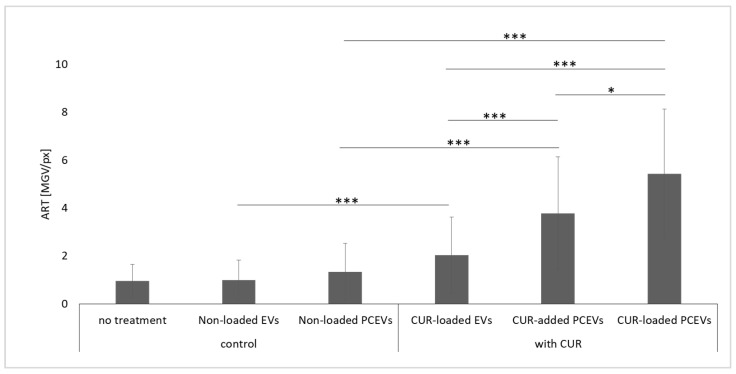
Dermal penetration efficacy of the produced formulations, which is derived from epifluorescence microscopy images subjected to an automated RGB threshold (ART) algorithm. Statistical comparisons were performed between the control formulations and their corresponding curcumin-based formulations. Also, curcumin-loaded EVs, curcumin-added PCEVs, and curcumin-loaded PCEVs were compared with each other. Significant differences between formulations are indicated in the figure with asterisks (*: *p* < 0.05, ***: *p* < 0.001).

Treatment with curcumin-loaded EVs was found to show the lowest penetration efficacy of 2 ± 2 [MGV/px], whereas curcumin-loaded PCEVs showed the highest efficacy of 5 ± 3 [MGV/px]. Furthermore, curcumin-loaded PCEVs achieved a 1.5-fold higher penetration efficacy compared to the curcumin-added PCEVs (*p* < 0.05) and demonstrated an around 2.7-fold t increase in penetration efficacy when compared to the curcumin-loaded EVs (*p* < 0.001) (Figure 6). These findings are in line with the data from the dermatokinetics studies and provide further evidence that curcumin-loaded PCEVs are an efficient nano-scaled drug carrier system to improve the dermal delivery of curcumin into the skin.

**Figure 7:**
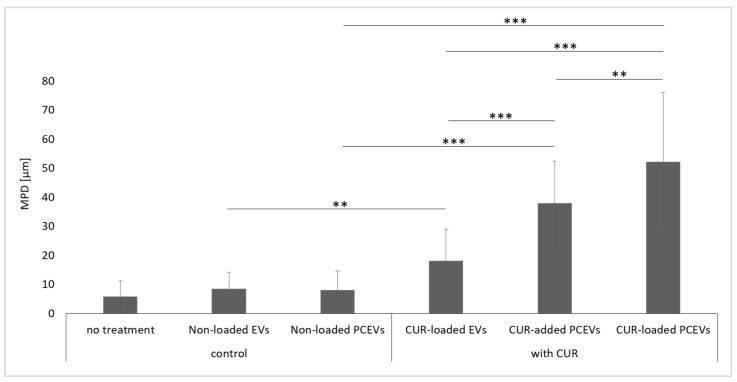
Mean penetration depth (MPD) [µm] of curcumin from different formulations. Statistical comparisons were performed between control formulations and their corresponding curcumin-based formulations. Also, curcumin-loaded EVs, curcumin-added PCEVs, and curcumin-loaded PCEVs were compared with each other. Significant differences between formulations are indicated in the figure with asterisks (**: *p* < 0.01, ***: *p* < 0.001).

**Figure 8:**
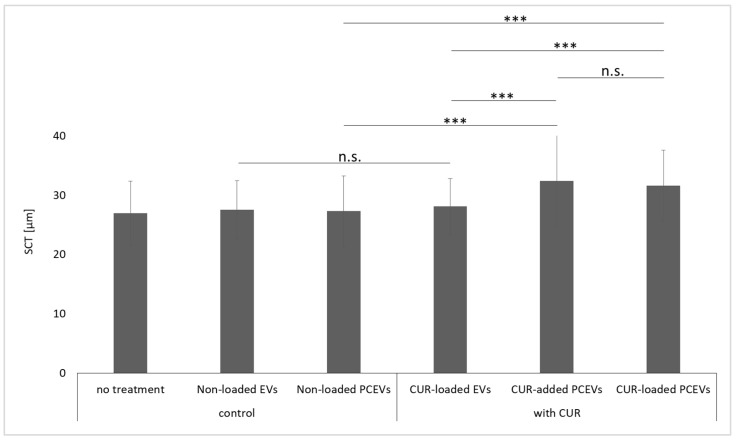
Stratum corneum thickness (SCT) [µm] of skin treated with different formulations. Statistical comparisons were performed between curcumin-loaded and non-loaded EVs (control), curcumin-added PCEVs, curcumin-loaded PCEVs, and non-loaded PCEVs (control), as well as between all curcumin-based formulations. Asterisks in the figure indicate significant differences between the groups (***: *p* < 0.001), and n.s.: indicates no significant difference.

The results of the mean penetration depth (MPD) for the investigated formulations are in line with the penetration efficacy data (Figure 7). Curcumin penetrated most deeply from the curcumin-loaded PCEVs and least deeply from the EVs. By comparing the MPD to the stratum corneum thickness (SCT) (Figure 8), it can be seen that curcumin from EVs could not penetrate through the stratum corneum, whereas curcumin penetrated through the stratum corneum (MPD > SCT) from the PCEVs. The superior penetration efficacy of PCEVs, when compared to EVs, can be due to different reasons. One main reason is considered to be the much larger particle size of EVs (>400 nm). In addition, polysorbate 80 is known to enhance the dermal penetration of poorly water-soluble drugs [47,48,49]. Its utilization in the production of plantCrystals production, but not in EVs, may have potentiated the enhanced curcumin dermal delivery observed with PCEVs compared to EVs. Other reasons could include variations in the zeta potential and differences in the interaction between curcumin and the skin, which may lead to variations in the diffusion coefficient of curcumin from the formulations into the skin. Further research is needed to understand these parameters in more detail. The improved penetration efficacy of the curcumin-loaded PCEVs when compared to the curcumin-added PCEVs was expected, and the results, therefore, provide evidence that efficient encapsulation of the active compounds into PCEVs, such as the addition of the active compounds to the PCEVs formulation prior to the milling process, is important for efficient dermal bioavailability.

In the next step, the influence of the formulations on the SCT was evaluated (Figure 8). The evaluation of SCT for treated skin can provide information about the hydration state of the stratum corneum and its changes upon different skin treatments [50,51,52]. No changes in SCT were found between untreated skin and the skin that was treated with unloaded EVs and PCEVs. However, the skin treated with curcumin-containing formulations showed increased an SCT. The reason is the hygroscopicity of curcumin [53,54]. This means that curcumin penetrates the stratum corneum, attracts water, and thus leads to an increase in SCT. Hence, the more curcumin penetrates the skin, the thicker the SCT. As the highest penetration of curcumin was seen from curcumin-loaded PCEVs, the most intense hydration of SCT was also seen in skin treated with this formulation. This means that curcumin-loaded PCEVs provide not only enhanced curcumin penetration but can also enhance skin hydration most efficiently.

**Figure 9:**
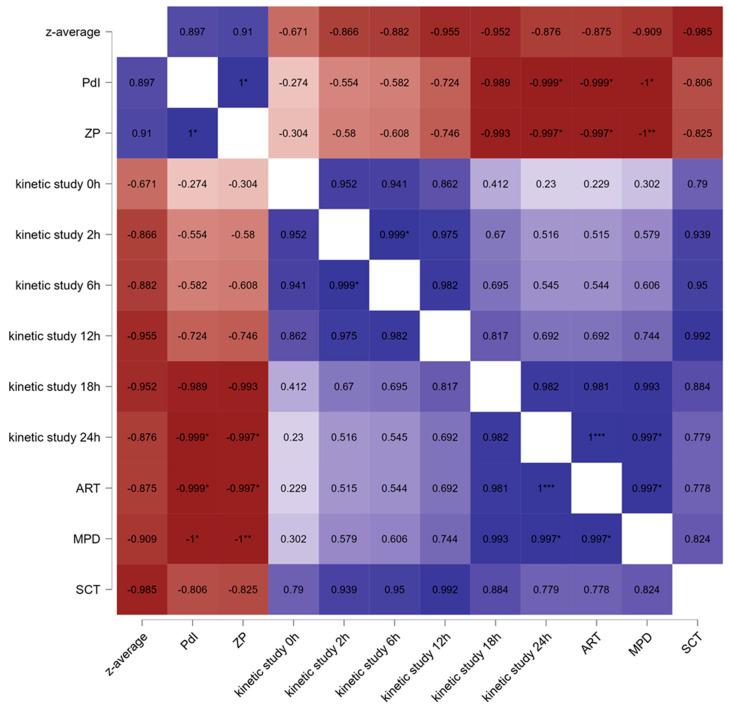
Pearson correlation illustrating the relationships between physicochemical properties, dermal penetration parameters (ART, MPD, and SCT), and dermatokinetic ex vivo porcine ear skin model for curcumin-loaded EVs. Particle size (z-average) and zeta potential (ZP). Significant differences are indicated by asterisks (* *p* < 0.05, **: *p* < 0.01 *** *p* < 0.001), while no asterisks denote no significant differences.

In the final step of this study, a correlation between the particle size (z-average), PdI, zeta potential, dermal penetration parameters (ART, MPD, and SCT), and the data obtained from the dermatokinetics studies was performed (Figure 9). Data show a positive correlation between particle size and PdI and zeta potential and confirm a negative correlation between particle size and penetration efficacy. In addition, a higher zeta potential was correlated with less efficient dermal penetration, which might be explained by the fact that the larger particles possessed a higher zeta potential. Interestingly, it was observed that larger particles led to a less hydrated stratum corneum. This is reasonable because a larger particle size means that fewer particles are present within these formulations, which means that fewer particles can interact with the skin. This reduced interaction with the skin results in less efficient hydration of the skin [38,44]. The correlation between the dermatokinetics data and the ex vivo penetration data (ART, MPD, and SCT) showed that the dermatokinetics data correlate best with the ex vivo model after 18 h. A reason for this is that the dermatokinetic model uses full-thickness skin and measures the amount of the active compound that penetrates through the skin. In the ex vivo model, the penetration into the skin but not through the skin is visualized. Therefore, the ex vivo model allows for faster discrimination between good and bad penetrating formulations. However, the SCT, which is a measure for the skin hydration, correlates best with the dermatokinetics data at earlier time points and shows the best correlation between 2 and 12 h. These data are of great interest because pig ears are known to undergo postmortem stiffness (rigor mortis) [55,56]. The onset of rigor mortis depends on many parameters and starts 4–6 h after slaughter. With the onset of rigor mortis, muscles contract, and water is squeezed out of the skin and hydrates the skin. Over time, water can evaporate and dry out of the skin, which results in a decreased SCT. The longer penetration times used in the dermatokinetics studies include the onset of rigor mortis and the drying out of the skin at later time points. It seems that the formulations that can hydrate the skin more efficiently prevent drying out of the skin due to rigor mortis, thereby leading to more efficient penetration and better correlation during these time points.

## 3. Materials and Methods

### 3.1. Materials

Curcumin, used as a BCS class IV model drug, was purchased from Receptura Apotheke (Cornelius-Apothekenbetriebs-OHG, Frankfurt am Main, Germany) in a dried form of *Curcuma longa* powder extract (purity = 95% curcuminoids, curcumin content 80%), soybeans were obtained frozen from a local supermarket in Marburg, Germany, and China was listed as the country of origin. Polysorbate 80, commercially known as Kolliphore^®^ 80, was obtained from BASF Pharma (Ludwigshafen am Rhein, Germany). Phosphate buffered saline (PBS, pH 7.4 at 25 °C) was used as the extraction buffer, and purified water was obtained using a PURELAB^®^ Flex 2 water purification system (ELGA Labwater, Veolia Water Technologies Deutschland GmbH, Celle, Germany).

### 3.2. Methods

#### 3.2.1. Preparation of Soybean-Derived Classical EVs

Frozen soybeans were weighed (total weight 50 g) and washed thrice with cold running tap water. An additional washing step was performed using purified water. The washed soybeans were then left to air dry at room temperature and peeled manually for their hull. The total weight of the soybeans after peeling was almost 45 g. The peeled defrosted soybeans were pre-ground manually for 5 min using a ceramic mortar and pestle. Filtered PBS (0.22 µm) was added to the ground soybeans to reach a final weight of 300 g, followed by grinding via an electrical grinder (Moulinex la moulinette ultimate dp810, Grenoble, France) for 5 min. Subsequently, the isolation of the soybean EVs was performed by differential ultracentrifugation (DUC), which included a series of low-velocity cycles at 400, 800, 2000, and 15,000× *g* using a Sorvall centrifuge (RC 6 Plus Centrifuge, Fisher Scientific GmbH, Schwerte, Germany) with a fixed angle rotor, each for 30 min at 4 °C. The pellets were discarded, and the resulting supernatants were collected. The supernatant was filtered using a 0.45 µm filter paper and centrifuged at 120,000× *g* for 60 min at 4 °C using a Sorvall ultracentrifuge (MTX 150 Micro-Ultracentrifuge, Fisher Scientific GmbH, Schwerte, Germany) with a S50-A fixed angle rotor [4,15,18,37]. The supernatants were carefully discarded without disturbing the pellet, as this last step was repeated, and fresh samples were added to the same tubes until all starting quantities were ultracentrifuged. The final isolate obtained was expected to contain soybean-derived classical EVs. It was suspended in a small volume of filtered PBS and vortexed vigorously for about 20 min.

#### 3.2.2. Preparation of Curcumin-Loaded EVs

The classical soybean-derived EVs were loaded by adding curcumin (0.5% (*w*/*w*)) to the blank classical EVs. The obtained samples were sonicated for 20 min, vortexed for 15 min, and incubated for 30 min at room temperature in the dark. This was followed by an additional ultracentrifugation step at 120,000× *g* for 60 min at 4 °C, in which the supernatant contained excess free curcumin particles that were not incorporated into the EVs, and the resultant pellet contained curcumin-loaded EVs [18,57].

#### 3.2.3. Preparation of Soybean-Derived PCEVs

Soybean-derived plantCrystals were produced using a small-scale bead milling method, as described previously, with slight modifications [18,22]. Before milling, the defrosted soybeans were washed, peeled, and manually ground with a ceramic mortar and pestle until a smooth paste was obtained. PCEVs were then produced using the bead milling process. Briefly, soybean paste was dispersed at a concentration of 1% (*w*/*w*) in a 1% (*w*/*w*) Kolliphore^®^ 80 surfactant solution. Yttrium-stabilized zirconium oxide beads (Ø 1 mm, SiLibeads^®^, Sigmund Lindner GmbH, Warmensteinach, Germany) were added at a 40:60 ratio of beads to soybean suspension, along with a magnetic stirring bar (Asteroid^®^ 25, 2 mag AG, München, Germany). This mixture was then placed in a 15 mL Erlenmeyer flask, which was set in an ice bath on a magnetic stirring plate (RCT Standard, IKA^®^ Werke GmbH & Co. KG, Staufen, Germany) and stirred at 1200 rpm for 8 h.

#### 3.2.4. Preparation of Curcumin-Added PCEVs and Curcumin-Loaded PCEVs

Curcumin-added PCEVs were produced by adding curcumin (0.5% (*w*/*w*)) to the produced unloaded plantCrystals dispersion. In addition, curcumin-loaded PCEVs were produced by adding curcumin to the mixture of soybean surfactant prior to the bead milling process, allowing curcumin to be incorporated into the PCEVs produced during the milling step.

#### 3.2.5. Physicochemical Characterization of the Formulations

The produced formulations were analyzed for their physicochemical properties. Particle size was determined by light Microscopy (LM), laser Diffraction (LD), and dynamic Light Scattering (DLS). The zeta potential (ZP) was also assessed to predict the physical stability of the produced formulations [18,40,58,59]. For LM analysis, the formulations were determined for their particle size and shape using an LM (Olympus BX53 microscope, Olympus Corporation, Tokyo, Japan) equipped with an SC50 CMOS color camera (Olympus soft imaging solutions GmbH, Münster, Germany). Agglomeration of the particles was also detected [38]. LD measurements were performed with a Mastersizer 3000 (Malvern-Panalytical, Kassel, Germany) to detect possible large particles within the PCEVs samples. Measurements (n = 10) were conducted with a HydroS dispersion unit and a stirring speed of 1750 rpm. Mie theory was applied for the analysis of the data with the optical parameters set to a real refractive index of 1.45 and the imaginary index set to 0.1 [18]. The results are expressed as the average values of volume-based diameters (d(v)0.10 − d(v)0.99) ± standard deviation. The DLS hydrodynamic particle size, PdI, and zeta potential measurements were all performed using the Zetasizer Nano ZS (Malvern-Panalytical, Kassel, Germany). For particle size and PdI measurements, the samples were diluted in purified water, and evaluation was performed using the general-purpose mode built into the software of the instrument. For zeta potential measurements, the samples were dispersed in conductivity-adjusted purified water (50 µS/cm). Zeta potential was calculated by measuring the electrophoretic mobility (EM) using Laser Doppler Anemometry. The measured EM was subsequently converted into zeta potential using the Helmholtz–Smoluchowski equation [38,60]. Measurements (n = 10) were conducted at 20 °C and are represented as average ± standard deviation.

#### 3.2.6. Dermal Penetration Efficacy of EVs and PCEVs

The dermal penetration efficacy of the curcumin-produced formulations was assessed using quantitative dermatokinetic and semi-quantitative ex vivo porcine ear models. These methods are described in detail below.

##### Assessment of Dermal Penetration Efficacy with the Quantitative Dermatokinetic Ex Vivo Porcine Ear Model

Fluorescent molecules can be detected across various wavelengths using a fluorescence plate reader, making them valuable for studies on the cellular uptake of fluorescent active compounds, such as curcumin [43,61,62]. Previous researchers adapted this approach along with the use of ex vivo porcine ear skin in a 24-well plate for the determination of penetration by measuring fluorescence intensity at the bottom of the well plate and assessing the dermal penetration kinetics of curcumin over 24 h [43].

**Figure 10:**
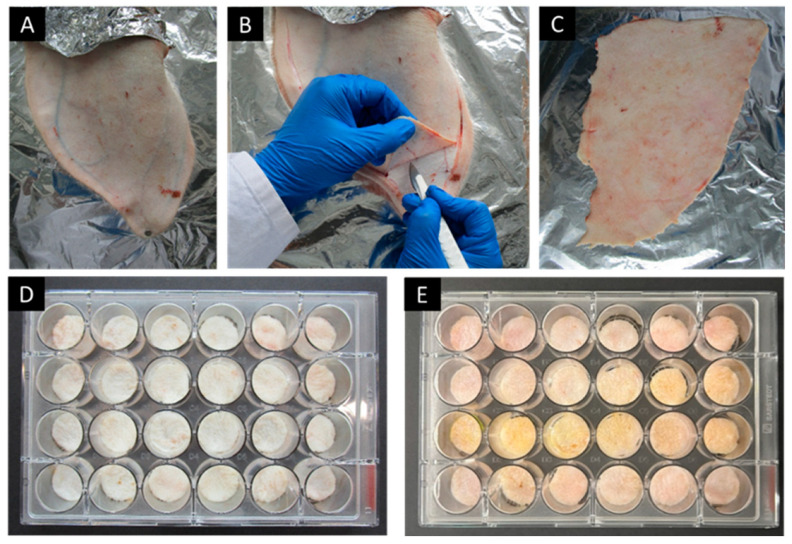
Overview of the experimental procedure for evaluating the dermal penetration kinetics of active compounds using a fluorescence plate reader; (**A**–**C**) dissection of the porcine ear skin from the upper portion of the cartilage, (**D**) placement of skin punches of 18 mm diameter into a well plate, and (**E**) application of formulations on the skin for further fluorescence intensity screening.

Briefly, the full thickness of the skin (around 1 mm thick) was obtained by removal of the skin from the dorsal side of the ex vivo porcine ear model, just above the upper cartilage, using a scalpel blade. Skin biopsies without any scratches or wounds were selected and punched with an (Ø 18 mm) punch. The biopsies were carefully placed in a covered 24-well plate and treated with 5 μL of the produced formulations, followed by incubation for 24 h at 32 °C. The samples were then analyzed using a fluorescence plate reader (Infinite^®^ 200 PRO; Tecan Group Ltd., Männedorf, Switzerland) (Figure 10). The fluorescence plate reader was set for bottom reading at specific excitation and emission wavelengths of curcumin, with excitation at 415 nm and emission at 570 nm. All measurements were performed in triplicate. The standard calibration curve was used to generate the regression Equation (1) to analyze the penetrated curcumin concentration, and the results were expressed as drug concentration in μg/mL at various time points over 24 h.

(y = 328604x + 10197, R^2^ = 0.9968), (1)

##### Evaluation of Dermal Penetration Efficacy Using the Ex Vivo Porcine Ear Model

Curcumin can be detected using inverted epifluorescence microscopy due to its own autofluorescence property [31]. The dermal penetration efficacy of curcumin-produced formulations was assessed utilizing the ex vivo porcine ear model and the digital image analysis to obtain the penetration parameters, i.e., the amount of penetrated curcumin (i.e., ART), MPD, and SCT. To obtain these parameters, steps were performed as described earlier with minor modifications [36,38,56]. First, fresh ears were obtained from a local slaughterhouse, cleaned with lukewarm water, and dried carefully with paper tissues. A skin area of 1.5 × 1.5 cm without visible scratches was selected. Prior to the application of the formulation, the hair in the selected skin areas was trimmed to a length of approximately 1–3 mm. Then, 15 µL was applied to the marked skin areas using the saturated glove finger technique to achieve a homogenous distribution of the formulations. The ears were incubated in an oven at 32 °C for 2 h to allow penetration of the formulation. Second, punch biopsies (Ø 15 mm) were taken and immediately embedded in Tissue-Tek^®^ (Sakura Finetek Europe B.V., Alphen aan den Rijn, The Netherlands) and frozen at −20 °C until further use. The study was conducted in triplicate using three different porcine ears. Third, the biopsies were collected and cut into 20 μm-thick vertical skin sections with a cryomicrotome (Frigocut 2700, Reichert-Junk, Nußloch, Germany), which were further subjected to inverted epifluorescence microscopy (Olympus CKX53) equipped with an Olympus DP22 color camera (Olympus Deutschland GmbH, Hamburg, Germany) to allow imaging of the skin sections. Forty images were obtained from each skin biopsy, allowing for 120 images to be collected for each formulation. The intensity of the fluorescent light source (130 W U-HGLGPS illumination system, Olympus Deutschland GmbH, Hamburg, Germany) and the exposure time were maintained constant at 50% and 50 ms, respectively. The settings were kept consistent throughout the analysis. The selected fluorescence filter for the analysis was the DAPI HC filter block system (excitation filter: 460–500 nm, dichroic mirror: 500 nm, emission filter: from 500 nm (LP)).

##### Digital Image Analysis

The penetration efficacy of curcumin from different produced formulations was assessed using ImageJ software according to previously established procedures [56,63,64]. The ART value for each formulation was determined, serving as a semi-quantitative measurement of penetrated curcumin. This value was evaluated by an automated threshold protocol that eliminated the autofluorescence of the skin (cf. Supplementary Table S1). In addition, the MPD of curcumin was assessed from the threshold images, and SCT was determined from the original images. The MPD and SCT measurements were evaluated using the scale function of the software. The scale was set to 2.84 px/µm.

#### 3.2.7. Statistical Analysis

The descriptive statistics of this section were also calculated using JASP software (version 0.18.3.0) (University of Amsterdam, Amsterdam, The Netherlands) [65]. Normality was assessed with the Shapiro–Wilk test, and variance homogeneity was evaluated using Levene’s test. Parametric data were analyzed using one-way analysis of variance (ANOVA). Post-hoc tests (Tukey, Games–Howell, or Dunn’s tests) were applied to check for significant differences between the mean values [66]. The Pearson correlation coefficient measures the linear relationship between data sets [67,68]. and was performed to assess the relationship between ART, MPD, and SCT and the penetration of curcumin in the dermatokinetic model at selected time points. The results are presented as mean values ± standard deviation. In the Figures, significant differences between mean values are denoted by * *p* < 0.05, ** *p* < 0.01, and *** *p* < 0.001, and standard deviations are expressed in error bars.

## 4. Conclusions

Soybean-derived EVs and PCEVs were successfully produced by classical isolation method and small-scale bead milling, respectively. Curcumin-loaded EVs, curcumin-loaded PCEVs, and curcumin-added PCEVs were also produced. EVs and PCEVs possessed different physicochemical properties, which resulted in differences in their dermal penetration efficacy for curcumin. The least efficient dermal penetration was found for the EVs. The best penetration and deepest penetration were found for the curcumin-loaded PCEVs. This provides evidence that PCEVs are promising drug delivery systems for curcumin and, most likely, other poorly water-soluble active compounds that belong to BCS class II or IV. Data also showed that the incorporation of curcumin into PCEVs is important. Hence, the active compound that needs to be delivered needs to be added during the production of PCEVs and cannot only be added after their production.

The correlation of the data showed a negative trend between particle size and dermal penetration efficacy, as well as between particle size and skin hydration. These trends are in line with previous studies and again prove the important influence of the physicochemical properties of the formulations on biopharmaceutical properties. Furthermore, the correlation data showed a positive correlation between the two different ex vivo models that were used for the assessment of dermal penetration efficacy and dermatokinetics studies. Both models provided valuable insights into the penetration behavior of curcumin into the skin from different carriers, and the combination of both models can be used to provide a thorough picture of the fate of curcumin and other active compounds after dermal application. In conclusion, the combination of both models is recommended as a powerful tool for testing the dermal penetration of active compounds from different formulations.

## Figures and Tables

**Table 1:** Zeta potentials of soybean-derived EVs and PCEVs with and without curcumin. Standard deviation (SD).

Formulation	Zeta Potential [mV] ± SD
Non-loaded EVs	−21 ± 2
CUR-loaded EVs	−18 ± 3
Non-loaded PCEVs	−35 ± 3
CUR-added PCEVs	−25 ± 1
CUR-loaded PCEVs	−30 ± 5

## Data Availability

Data are contained within the article.

## Supplementary Materials

The following supporting information can be downloaded at: https://www.mdpi.com/article/10.3390/molecules29245926/s1, Table S1: Macro used for the automated RGB threshold for the digital image analysis.
