# Supplementary material for: Extracellular Vesicles and PlantCrystals for Improved Bioavailability of Curcumin as a BCS Class IV Drug
Source: Molecules. 2024 Dec 16;29(24):5926. doi: 10.3390/molecules29245926 (PMC11677860; doi:10.3390/molecules29245926)
Supplement: Supplementary file 1 [file molecules-29-05926-s001.zip › molecules-3355287-supplementary.pdf]

Extracellular Vesicles and PlantCrystals for Improved Bioavailability of Curcumin as a BCS Class IV Drug

Muzn Alkhaldi [1], Tehseen Sehra [1,2], Soma Sengupta [1] and Cornelia M. Keck [1,*]

[1] Department of Pharmaceutics and Biopharmaceutics, Philipps-Universität Marburg, Robert-Koch-Str. 4, 35037 Marburg, Germany; muzn.alkhaldi@pharmazie.uni-marburg.de (M.A.); tehseen.sehra@pharmazie.uni-marburg.de (T.S.); sengupts@pharmazie.uni-marburg.de (S.S.)

[2] Institute of Pharmacy, Faculty of Pharmaceutical and Allied Health Sciences, Lahore College for Women University, Lahore, 54000, Pakistan

* Correspondence: cornelia.keck@pharmazie.uni-marburg.de

Table S1: Macro used for the automated threshold to subtract the autoflorescence of the skin from the fluorescence of the curcumin penetrated

```
// Color Thresholder 1.53k
// Autogenerated macro, single images only!
min=newArray(3);
max=newArray(3);
filter=newArray(3);
a=getTitle();
run("RGB Stack");
run("Convert Stack to Images");
selectWindow("Red");
rename("0");
selectWindow("Green");
rename("1");
selectWindow("Blue");
rename("2");
min[0]=0;
max[0]=0;
filter[0]="stop";
min[1]=66;
max[1]=255;
filter[1]="pass";
min[2]=0;
max[2]=0;
filter[2]="stop";
for (i=0;i<3;i++){
  selectWindow(""+i);
  setThreshold(min[i], max[i]);
  run("Convert to Mask");
  if (filter[i]=="stop")  run("Invert");
}
imageCalculator("AND create", "0","1");
imageCalculator("AND create", "Result of 0","2");
for (i=0;i<3;i++){
  selectWindow(""+i);
  close();
}
selectWindow("Result of 0");
close();
selectWindow("Result of Result of 0");
rename(a);
// Colour Thresholding-------------
run("Invert");
```
